# Shengyang Yiwei Decoction for the treatment of chronic gastritis: A protocol for a systematic review and meta-analysis

**DOI:** 10.1097/MD.0000000000022869

**Published:** 2020-10-23

**Authors:** Min Xiong, Huan Luo, Wenyu Zhu, Tao Shen

**Affiliations:** College of basic medicine, Chengdu university of Traditional Chinese Medicine, Chengdu, Sichuan, China.

**Keywords:** chronic gastritis, protocol, randomized controlled trial, shengyang yiwei decoction, systematic evaluation, traditional chinese medicine

## Abstract

**Background:**

Chronic gastritis is a very common chronic gastric mucosal inflammatory disease in China. Long-term chronic inflammation will aggravate the disease and develop towards gastric cancer. Clinically, infection with *Helicobacter pylori* (*H pylori)* is a common cause. The application of antibiotics to eradicate *H pylori* has been reported to have produced drug resistance. However, the application of Shengyang Yiwei Decoction(SYD) in traditional Chinese medicine has resulted in significant clinical effects and small side effects. It is used for the treatment of chronic gastritis and other diseases, but there is a lack of systematic reviews on the treatment of chronic gastritis by SYD. This article reviews the efficacy and safety of SYD in the treatment of chronic gastritis.

**Methods:**

The registration date for the randomized controlled trials is set by the database to October 15, 2020. Through searching the following 8 Chinese and English electronic databases: Cochrane Library, Embase, PubMed, Science Net, China National Knowledge Infrastructure, Chinese Biomedical Literature Database, Chinese Science Journal Database and Wanfang Database to analyze. The main results were clinical efficacy, *H pylori* infection clearance rate, symptom score and quality of life, and 1-year recurrence rate. Finally, Stata 15 was used for meta-analysis.

**Results:**

This study will provide the latest evidence for the treatment of chronic gastritis by SYD in the following aspects: clinical efficacy, *H pylori* infection clearance rate, quality of life, symptom score, and 1-year recurrence rate.

**Conclusion:**

The results of this study will provide evidence for evaluating the effectiveness of SYD in chronic gastritis treatment.

**OSF registration number:**

DOI 10.17605/OSF.IO/AZKUQ

## 1 Introduction

As a chronic inflammatory disease, chronic gastritis (CG) is the most common among the general population in China. It is divided into non-atrophic gastritis and atrophic gastritis,^[1]^ among which Helicobacter pylori (*H pylori)* infection is 1 of the causes of atrophic gastritis, *H pylori* Gastritis increases the risk of cancer associated with atrophy and intestinal metaplasia.^[2]^ In the monitoring of precancerous lesions of gastric cancer, patients with intestinal metaplasia are at high risk.^[3]^ atrophic gastritis patients within 5 years after diagnosis of gastric cancer in incidence was 0.1%,^[4]^ The application of antibiotics to eradicate *H pylori* in treatment has greatly reduced the risk of cancer and has a significant early effect. However, the eradication of *H pylori* has failed due to its resistance to antibiotics,^[5]^ the postoperative recurrence rate of endoscopic mucosal resection for early gastric cancer Higher.^[6]^ The development and application of traditional Chinese medicine (TCM) has opened a new milestone in the treatment of CG. A large number of clinical studies^[7–10]^ have shown that TCM has played a key role in the treatment of CG and its development into gastric cancer.

Shengyang Yiwei Decoction (SYD) is a “prescription for spleen and stomach deficiency of the lung” in the volume of “Distinguishing the Confusion of Internal and External Injury” by Li Gao during the Jin and Yuan Dynasties. It mainly treats the symptoms of spleen and stomach weakness, damp-heat internal depression, and is useful in the treatment of chronic atrophic gastritis. Obvious curative effect.^[11–14]^ This product is composed of astragalus, white peony root, coptis, ginseng, atractylodes, tuckahoe, licorice, tangerine peel, pinellia, Qianghuo, single living, and windproof. The Liujunzi Decoction contained in it is a commonly used prescription for the treatment of spleen and stomach. Liujunzi Decoction is used to invigorate qi and invigorate the spleen and reduce dampness. It is combined with astragalus to promote Yang Qi, Qianghuo, Duhuo, Fangfeng promote clearing Yang, expel wind and dampness, and white peony nourishes blood and promotes health. It has a good effect on anorexia, hiccups, acid reflux, nausea and vomiting in the gastrointestinal tract.

Pharmacological studies have shown that ginseng and astragalus have a regulatory effect on chronic atrophic gastritis,^[15]^ coptis has a significant anti-inflammatory effect,^[16]^ and the polysaccharides and poria cocos in Poria cocos have anti-tumor, immune regulation, anti-inflammatory, and hypoglycemic properties. A variety of biological activities such as lowering blood lipids,^[17]^ Bupleurum, Fangfeng, Duhuo, licorice, ginger have anti-inflammatory and analgesic effects.^[18]^ A large number of recent clinical studies have shown that SYD has a significant effect on the treatment of CG. There is an urgent need for a systematic review to support the effectiveness and safety of SYD in the treatment of CG. Therefore, the purpose of this study is to systematically review the existing literature to evaluate the effectiveness and safety of SYD in the treatment of CG patients.

## 2 Methods

### 2.1 Protocol and registration

The scheme was formulated according to the preferred reporting items of the Guidelines for Systematic Review and Meta-Analysis. It has been registered on the Open Science Framework platform (https://osf.io/azkuq), the registration number: DOI 10.17605/OSF.IO/AZKUQ

### 2.2 Types of studies

Randomized controlled clinical trials published in English will be selected, whether blind or not. Without time constraints.

### 2.3 Types of patients

Participants must meet the diagnostic stan- dard of the second national consensus on CG will be involved irrespective of their age, ethnicity, or sex. Patients with other complicating diseases will will be excluded.

### 2.4 Types of interventions

In treatment group, SYD will be the sole treatment for patients, while routine western medicines will be used alone in control group.

### 2.5 Outcomes

The following main outcomes will be measured: clinical efficiency, *H pylori* infection clearance rate, quality of life and symptom scores. The second is the 1-year recurrence rate.

### 2.6 Search strategy

#### 2.6.1 Electronic searches

From the establishment of each database to October 2020, a comprehensive search will be conducted through the following electronic databases:Cochrane Library, Embase, PubMed, Web of Science, Chinese Scientific Journal Database, China National Knowledge Infrastructure, China Biomedical Literature Database, and Wanfang Database. Key words include “Shengyang Yiwei Decoction”, “chronic gastritis”, etc. The initial search methods of PubMed are shown in Table 1. Relevant data will also be searched through other sources.

**Table 1:**
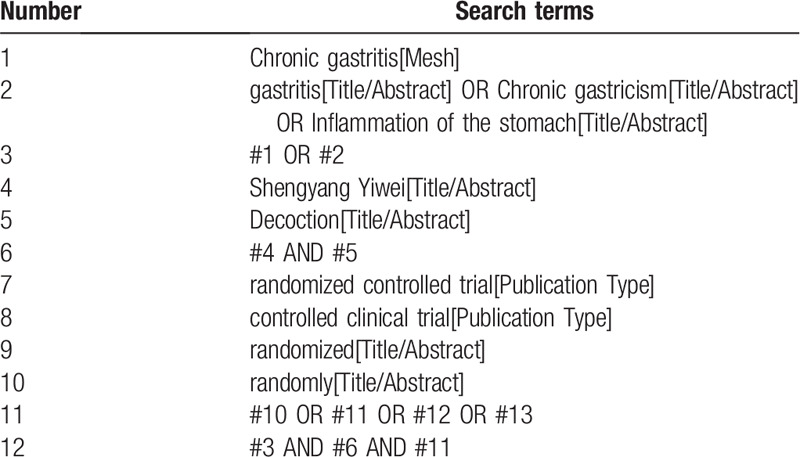
Search strategy of the PubMed.

### 2.7 Data collection and analysis

#### 2.7.1 Selection of studies

**Figure 1:**
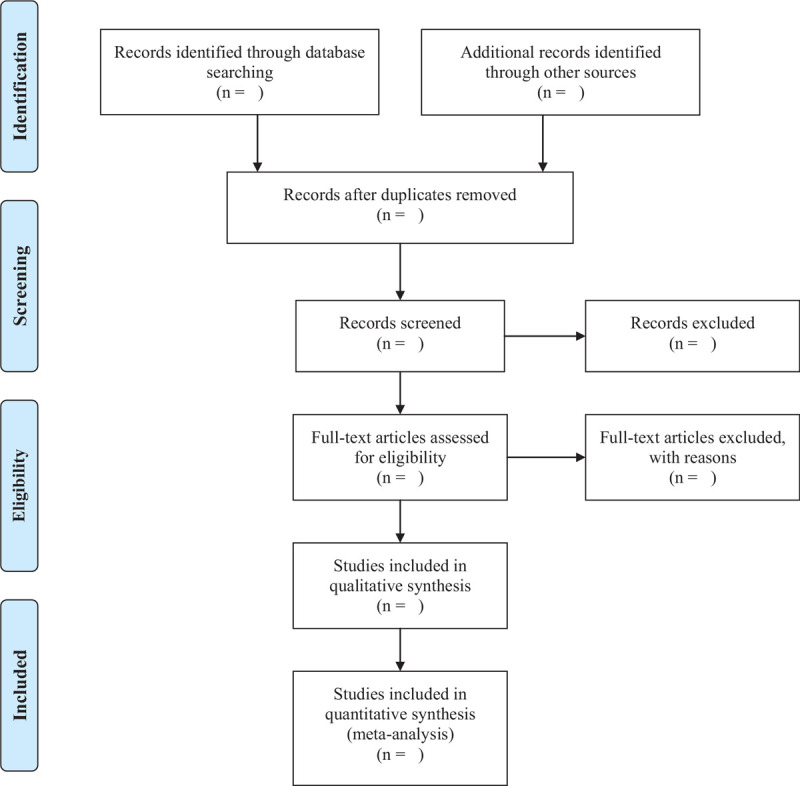
PRISMA flow diagram of the study selection process. PRISMA = preferred reporting items of the guidelines for systematic review and meta-analysis.

As shown in Figure 1, 2 researchers (Min Xiong and Wenyu Zhu) imported the retrieved documents into Endnote X9 software for review, deleted duplicate references, and initially screened the abstract to exclude documents that obviously did not meet the inclusion criteria. Then, Download and read the full text for follow-up inspection. After the final inclusion, we will use the pre-designed data extraction table to extract data and cross-check the results. If there is any objection, we will ask a third researcher (Huan Luo) to assist in making a judgment.

#### 2.7.2 Data extraction

Data extraction will be conducted by 2 researchers (Huan Luo and Wenyu Zhu) in a standardized abstract form of data, consisting of 4 parts: basic information of the study, characteristics of the test subjects, intervention measures, research results and measurement data. Similarly, if there is any discrepancy, the final decision will be made by the third reviewer (Tao Shen). If there is missing or insufficient information, we will analyze and deal with it, and consider the impact of missing data on the meta-analysis results.

#### 2.7.3 Quality evaluation on methodology

According to the Cochrane “system - ventions v. 5.2.0 system evaluation handbook (update) in June, 2017 standards, including 7 domains: random distribution method is hidden and blind patients, dazzling the results of the assessment, incomplete data processing results (ie, whether to describe the subsequent losses, the number of the export, whether intentionally analysis), selective reports, other bias. The quality of the included literature will be divided independently by 2 researchers into 3 categories (low risk,high risk,and unclear). If there is disagreement, consensus will be reached through discussion or consultation with the third author.

#### 2.7.4 Statistic analysis

Meta-analysis was performed using Stata 15. The results of the dichotomy will be expressed as the relative risk or odds ratio of the 95% confidence interval (CI). For continuous outcomes, if the outcome measurements of alzl studies are based on the same measurement unit, then the mean difference of 95% CI is given; otherwise, the standard mean difference of 95% CI is given for analysis.

#### 2.7.5 Assessment of heterogeneity

Statistical heterogeneity between studies will be assessed by *I*^2^ and chi-square statistics. If *I*^2^ is between 50% and 100%, there is a statistical heterogeneity, for which we will use a random effects model to analyze the data. If the heterogeneity test is not significant (*I*^2^≤50%), the fixed-effect model is used. In addition, due to differences in heterogeneity, we will conduct subgroup or sensitivity analysis to look for potential causes.

#### 2.7.6 Assessment of reporting bias

Funnel plots will be drawn to assess report bias. If a potential reporting basis is found, the Berg and Egger tests will be used to assess funnel plots for symmetry and perceived publication bias.

#### 2.7.7 Sensitivity analysis

Sensitivity analysis was used to evaluate the robustness of the main efficacy indicators. The method was to eliminate the low-quality studies 1 by 1, merge the data, and evaluate the impact of sample size, research quality, statistical methods and missing data on the meta-analysis results.

#### 2.7.8 Subgroup analysis

If the results are heterogeneous, we will conduct a subgroup analysis based on different reasons. Heterogeneity is mainly manifested in race, age, gender, drug dosage form, different forms of intervention, course of treatment, doseand other aspects.

#### 2.7.9 Quality of evidence

The reliability of the evidence will be assessed by grading recommendations for evaluation, development, and evaluation. The quality of evidence will be classified as high, medium, low or very low.

#### 2.7.10 Ethics and dissemination

Our goal is to publish this review in a peer-reviewed journal. Private information from individuals is not subject to review and therefore no informed consent is required. Ethical approval is also unnecessary because the study is not a clinical trial. Patients and the public did not participate.

## 3 Discussions

CG is a common chronic gastric mucosal inflammatory disease. Its incidence ranks first among various gastric diseases. It is divided into non-atrophic gastritis and atrophic gastritis. *H pylori* infection is a common cause of atrophic gastritis. Damage to mucosal function will not only affect the quality of life of patients, but will also increase the risk of cancer under the stimulation of long-term inflammatory factors and other harmful substances. The current treatment methods rely on eradication of *H pylori* to achieve poor cure results, and the recurrence rate after surgical treatment is high. The advantages of TCM in the treatment of CG are increasingly emerging, which can not only relieve the pain of patients from the symptoms, but also fundamentally solve the patients’ pain. The cause of the disease is to seize the pathogenesis to adjust the patient's physique, enhance the body's transport, chemistry, and transport functions. Modern pharmacological studies have also confirmed that SYD has a definite pharmacological effect on chronic atrophic gastritis, and has obvious anti-inflammatory, anti-tumor, and hypoglycemic effects. However, the systematic review of SYD treatment of CG has not been published yet. This systematic review will be the first to provide a summary of the current status of evidence regarding the effectiveness and safety of SYD in the treatment of CG. This assessment will help doctors and patients’ CG.

## Author contributions

Min Xiong and Wenyu Zhu made similar contributions to literature retrieval and research, and wrote the first draft of the agreement. Min Xiong developed a search strategy. Huan Luo, Min Xiong and Wenyu Zhu will conduct literature retrieval and collation. Huan Luo, Min Xiong and Tao Shen will assess the risk of bias in the literature. Data analysis and article writing will be completed by Min Xiong and Huan Luo. As corresponding author, Shen Tao will be responsible for supervising every process of audit and controlling the quality of research. All the authors approved the publication of the program.

**Data curation:** min xiong, huan luo.

**Formal analysis:** min xiong, wenyu zhu.

**Project administration:** min xiong.

**Software:** min xiong, huan luo, wenyu zhu.

**Validation:** min xiong, tao shen, huan luo.

**Writing – original draft:** min xiong, huan luo.

**Writing – review & editing:** min xiong, wenyu zhu.

## References

[1] Rugge M Genta RM Staging and grading of chronic gastritis. Hum Pathol 2005;36:228–33. 15791566 10.1016/j.humpath.2004.12.008

[2] Ben Slama S Ben Ghachem D Dhaoui A Gastrites chroniques à hélicobacter pylori: évaluation des systèmes OLGA et OLGIM [Helicobacter pylori gastritis: assessment of OLGA and OLGIM staging systems]. Pan Afr Med J 2016;23:28. 27200133 10.11604/pamj.2016.23.28.8839 PMC4856514

[3] den Hoed CM Holster IL Capelle LG Follow-up of premalignant lesions in patients at risk for progression to gastric cancer. Endoscopy 2013;45:249–56. 23533073 10.1055/s-0032-1326379

[4] de Vries AC van Grieken NC Looman CW Gastric cancer risk in patients with premalignant gastric lesions: a nationwide cohort study in the Netherlands. Gastroenterology 2008;134:945–52. 18395075 10.1053/j.gastro.2008.01.071

[5] Wei W Yang Y Diagnosis and treatment of chronic atrophic gastritis and advantages of traditional Chinese medicine. J Trad Chin Med 2016;57:36–40.

[6] Lian Y Enqiang L Zhiqiang W Investigation on gastric intraepithelial neoplasia and recurrence rate after endoscopic mucosal resection (EMR) for early cancer. Chinese journal of continuing medical education 2011;14:101–3.

[7] Dong Y Professor Yao Naili's academic thoughts on the treatment of spleen and stomach diseases and clinical research on the Differential treatment experience of chronic gastritis. Chinese Academy of Chinese Medical Sciences 2016.

[8] Beijing University of Chinese Medicine, Yanhong L Clinical Efficacy observation of ”Wenyang Jianpi Decoction" in the treatment of chronic atrophic gastritis with spleen and stomach weakness. 2016.

[9] Beijing University of Chinese Medicine, Nin L Academic experience of Professor Jingyuan Liu and clinical study on the treatment of chronic atrophic gastritis (syndrome of spleen-stomach weakness) with flavored-Astragalus Jianzhong. 2016.

[10] Beijing University of Chinese Medicine, Hongmei G Zeng Bin Fang's academic thoughts and clinical experience and The clinical study of Fuzheng Xiaowei Decoction in the treatment of chronic atrophic gastritis with spleen and stomach weakness. 2016.

[11] Xiaoyan L Juanping S Wujin F Preliminary study on the therapeutic effect of shengyang yiwei decoction on chronic atrophic gastritis with spleen-stomach weakness. Chinese folk therapy 2019;27:

[12] Shaomei S Guiling S Xuemei C Shengyang yiwei decoction for the treatment of 86 cases of chronic atrophic gastritis. Chinese journal of practical medicine 2019;14:133–4.

[13] Jinzhou Medical University, Jia C Clinical efficacy and serum proteomics of Shengyang Yiwei Decoction in the treatment of chronic atrophic gastritis with spleen-stomach weakness. 2017.

[14] Jia C The Second Qing Dynasty. Effect of shengyang yiwei decoction on spleen-stomach weakness of chronic atrophic gastritis. Chinese modern applied pharmacy 2016;33:1056–9.

[15] Weihan Z Rui S Meijuan Y Effects of Astragaloside IV and ginsenoside Rg1 on the regulation of Hedgehog signaling pathway in rats with chronic atrophic gastritis. Global Chin Med 2017;10:1428–33.

[16] Guangzhou University of Chinese Medicine, Xiuting Y Discussion on the role and mechanism of the main active components of Patchouli and Coptis chinensis against inflammatory bowel disease. 2016.

[17] Nian Z Zhaoxing L Juan L Advances in the study of chemical composition and bioactivity of poria cocos. World science and technology - modernization of traditional Chinese medicine 2019;21:220–33.

[18] Gansu University of Traditional Chinese Medicine (formerly known as Gansu College of Traditional Chinese Medicine), Yiwei Z Effects of Shengyang Yiwei Decoction on Th1/Th2 balance and EGF expression in rats with chronic atrophic gastritis (CAG). 2015.

